# Enhanced electromagnetic wave absorption performance by introducing exchange bias in a CIP@γ-FeOOH heterostructure

**DOI:** 10.1016/j.isci.2026.114822

**Published:** 2026-01-29

**Authors:** Yunpeng Li, Luyang Li, Haojie Zhang, Jixing Bai, Lihong Gao, Zhuang Ma, Qi Cao, Miao Jiang

**Affiliations:** 1 School of Materials Science and Engineering, Beijing Institute of Technology, Haidian, Beijing 100081, P.R. China; 2 National Key Laboratory of Science and Technology on Materials under Shock and Impact, Beijing Institute of Technology, Haidian, Beijing 100081, P.R. China; 3 Materials Intelligent Innovation Laboratory (MIIL), Beijing Institute of Technology, Zhuhai, Guangdong 519088, P.R. China; 4 Yangtze Delta Region Academy in Jiaxing, Beijing Institute of Technology, Jiaxing, Zhejiang 314000, P.R. China; 5 Key Laboratory of Energy Thermal Conversion and Control of Ministry of Education, Key Laboratory of Functional Polymers for Sustainability of Jiangsu Province, School of Energy and Environment, Southeast University, Nanjing 210096, China

**Keywords:** Physics, Engineering, Materials science

## Abstract

High-performance electromagnetic wave (EMW) absorbing materials are urgently needed to mitigate electromagnetic (EM) pollution. Carbonyl iron powder (CIP), a conventional magnetic loss absorber, often shows limited absorption bandwidth at low filler loadings. Here, we report a transition-layer-guided oxidation strategy to construct a hierarchical CIP@γ-FeOOH core-shell absorber. A sacrificial SiO_2_ shell enables the uniform growth of a flower-like γ-FeOOH shell while protecting the CIP core. The resultant ferromagnetic/antiferromagnetic (FM/AFM) interface induces a significant exchange bias effect, enhancing magnetic loss via interfacial pinning and strengthening the low-frequency magnetic response. Combined with defect-induced polarization and structural scattering, the composite breaks the Snoek’s limit constraint with a maximum effective absorption bandwidth (EAB_max_) of 6.13 GHz (9.38–15.51 GHz) and a minimum reflection loss (*RL*_min_) of −17.68 dB at 60 wt. %. These findings provide a structural and mechanistic basis for designing CIP-based EMW absorbers with improved broadband performance through interfacial engineering.

## Highlights

• Sacrificial SiO_2_ layer regulates oxidation to form CIP@γ-FeOOH
• FM/AFM interface induces exchange bias to boost low-frequency magnetic loss
• CIP@γ-FeOOH breaks Snoek’s limit with broadband EMW absorption

## Graphical abstract

**Figure:**
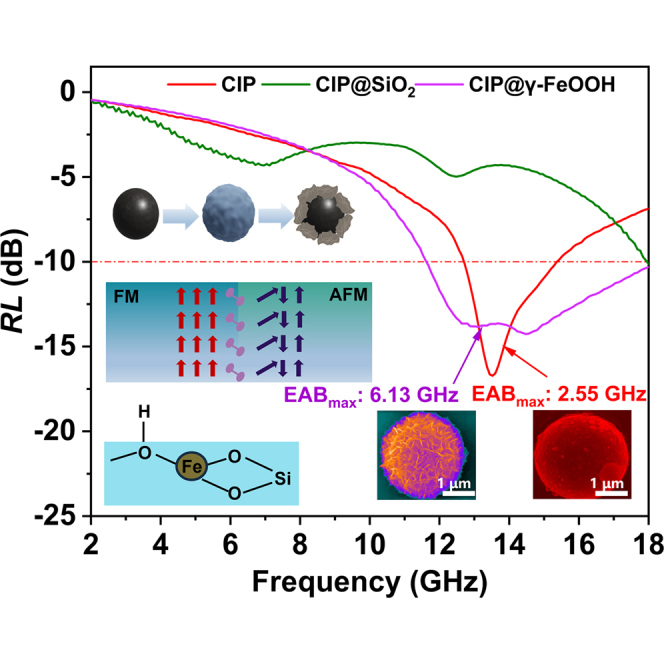

## Introduction

High-performance electromagnetic wave (EMW) absorbing materials are urgently needed to mitigate escalating electromagnetic (EM) pollution.^1^^,^^2^^,^^3^^,^^4^^,^^5^ Carbonyl iron powder (CIP), known for its strong magnetic loss, is a leading candidate. However, its performance at low filling ratios is fundamentally constrained by poor impedance matching and a narrow absorption bandwidth, creating a critical bottleneck for advanced applications.^6^^,^^7^^,^^8^^,^^9^^,^^10^^,^^11^^,^^12^

The pursuit of optimal EMW absorption performance in magnetic absorbing materials necessitates simultaneous high magnetic loss and well-matched impedance. Current core-shell interface designs often address only one aspect of this requirement. Strategies aimed at boosting magnetic loss, such as Fe core geometric modulation^13^ or Fe/Fe_3_O_4_ interface construction,^14^ typically do not resolve inherent impedance mismatch. Conversely, approaches designed to improve impedance matching, like coating with low-permittivity (*ε*_r_) oxides (SiO_2_, ZnO, TiO_2_)^15^^,^^16^^,^^17^ or high-conductivity C layers,^18^^,^^19^^,^^20^ inherently dilute magnetic content, and face challenges in precise thickness control (deviation>20 nm).^16^^,^^18^^,^^20^ This inability to concurrently optimize both properties hinders the synergistic effect needed to transcend Snoek’s limit for broadband performance.

The exchange bias effect at ferromagnetic/antiferromagnetic (FM-AFM) interfaces demonstrates unique EM parameter modulation capabilities, offering novel design principles for functional materials.^21^ Che et al.^22^ established an FM-AFM coupled system through Ni-NiO core-shell structures, where interfacial pinning effects from NiO significantly enhanced permeability (*μ*_r_) and impedance matching. This breakthrough highlights the untapped potential of systematically engineering AFM materials within CIP systems for broadband low-frequency(*f*) absorption. While conventional oxides such as MnO^23^ and Fe_2_O_3_^24^^,^^25^ dominate current investigations, iron oxyhydroxides (FeOOH) with concurrent AFM characteristics, high defect density, and proton conduction properties remain underexplored in EMW applications.^26^^,^^27^ Existing synthesis routes predominantly rely on Fe^2+^/Fe^3+^ precursors,^28^^,^^29^^,^^30^^,^^31^^,^^32^^,^^33^ and they lack systematic protocols compatible with CIP materials. Crucially, precise control over iron oxidation kinetics and the stability-activity tradeoff in interfacial pinning effects remain formidable technological barriers.

Herein, CIP@γ-FeOOH heterostructures were prepared using a transition-layer-guided oxidation method. The oxidation kinetics were modulated by introducing a SiO_2_ shell, enabling the formation of a uniform γ-FeOOH shell while protecting the core from excessive etching. By forming interfaces between FM and AFM regions, the method enhances magnetic coupling, which in turn leads to a higher imaginary part of permeability (*μ*″). The γ-FeOOH shell introduces defect-driven polarization and enhances multi-scattering. We uncover how the combined dielectric and magnetic response surpasses the Snoek limit and fine-tunes impedance matching in the X-band.

## Results and discussion

### Morphology and structural characterizations

**Figure 1:**
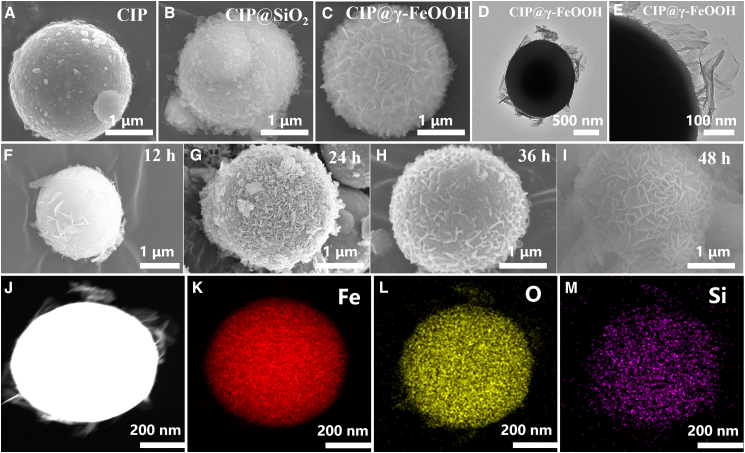
Morphological and microstructural characterization of CIP-based materials SEM images of (A) CIP, (B) CIP@SiO_2_, (C) CIP@γ-FeOOH; HR-TEM images of CIP@γ-FeOOH (D and E); schematic illustration of γ-FeOOH growth on CIP at 8 h (F), 16 h (G), 24 h (H), and 48 h (I); TEM surface scans of CIP@γ-FeOOH (J–M).

The morphological and interfacial evolution of the CIP-based powders during the controlled oxidation process is systematically presented in Figure 1. After the sol-gel deposition of the SiO_2_ shell, the CIP surface becomes decorated with loosely distributed nanoparticles, as shown in Figures 1A and 1B. During subsequent alkaline hydrothermal treatment, the SiO_2_ shell partially dissolves, generating local alkaline microenvironments and additional reactive sites. Meanwhile, the oxidation of the CIP proceeds through the remaining SiO_2_ channels, and the outward diffusion of Fe^3+^ ions under spatial confinement leads to the extrusion and anisotropic growth of lamellar γ-FeOOH structures, as shown in Figure 1C. The SiO_2_ transition layer provides chemical inertness and a conformal interface, preventing rapid and uncontrolled corrosion of the CIP surface during hydrothermal oxidation. Without this protective layer, CIP undergoes direct, aggressive surface oxidation, leading to irregular, rough, and discontinuous FeOOH deposits instead of a uniform shell, as shown in Supporting Information S1. HR-TEM imaging (Figures 1D and 1E) captures the *in-situ* nucleation of lamellar structures, and Figures 1F–1I schematically illustrate the four-stage evolution over hydrothermal durations of 8, 16, 24, and 48 h, respectively. As the process continues, Fe^2+^/Fe^3+^ species not only react with OH^−^ to form FeOOH nuclei but also compete with the silicate species for hydroxyl coordination, gradually substituting Si atoms and promoting the complete transformation of the surface into γ-FeOOH. With increasing reaction time, these nanosheets densify and self-organize into a hierarchical flower-like architecture. High-resolution TEM (Figures 1J–1M) reveals that the resulting shell consists of loosely packed γ-FeOOH lamellae with embedded Si–O coordination species, forming a heterogeneous, porous interface. This multi-stage interfacial reconstruction demonstrates that the transient SiO_2_ shell plays a dual role—retarding the oxidation kinetics of CIP while providing both physical confinement and chemical mediation for the anisotropic formation of the γ-FeOOH shell.

**Figure 2:**
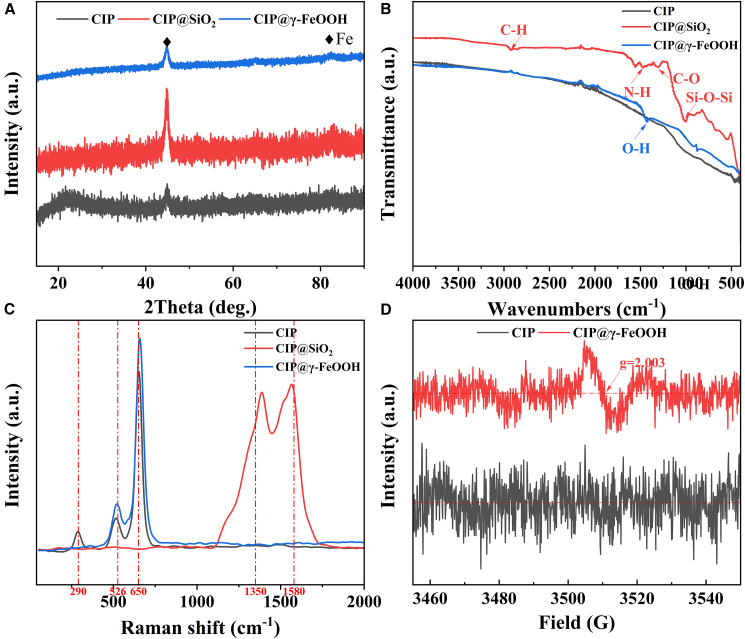
Structural and spectroscopic characterization of CIP-based materials (A) XRD patterns. (B) FTIR spectra. (C) Raman spectra. (D) EPR spectra.

A series of detailed characterizations is conducted to unravel the compositional transitions and underlying interfacial bonding mechanisms. XRD patterns in Figure 2A reveal no discernible phase transition during surface modification. All samples exhibit characteristic peaks at 2θ = 44.35°, corresponding to the (110) planes of Fe (JCPDS No. 06–0696).^34^ The absence of new diffraction signals indicates that both the SiO_2_ and γ-FeOOH layers remain predominantly amorphous. FTIR spectra, as shown in Figure 2B, further illuminate the evolution of surface chemical environments. Weak features at around 2920 cm^−1^, 1560 cm^−1^, and 1320 cm^−1^ are indicative of residual KH550 organosilane introduced during the SiO_2_ synthesis, and which align with expected overtone/combination vibrations of C–H, N–H, and C–O groups.^35^ The CIP@SiO_2_ sample displays a broad asymmetric band at ∼1020 cm^−1^, attributed to the Si–O–Si stretching mode in amorphous SiO_2_.^36^ This vibrational signature disappears after hydrothermal treatment, accompanied by the emergence of a distinct band at 1427 cm^−1^, which corresponds to –OH bending vibrations.^37^ The presence of this new signal that is absent in both CIP and CIP@SiO_2_ may indicate the *in situ* formation of hydroxyl-rich FeOOH layers via controlled oxidation. Raman spectroscopy (Figure 2C) provides further insight into the chemical identity and electronic environment of surface species. Pristine CIP exhibits a low-intensity A_1_g mode at 290 cm^−1^, indicative of Fe_2_O_3_ (goethite).^38^ In contrast, CIP@γ-FeOOH displays characteristic double peaks at ∼526 cm^−1^ (B_1_g) and ∼650 cm^−1^ (A_1g_), consistent with γ-FeOOH (lepidocrocite).^39^ Notably, these Raman bands are blue-shifted relative to standard γ-FeOOH positions, implying reduced electron density near the Fe centers, possibly due to surface deprotonation or altered ligand field strength.^40^ Inversely, CIP shows red-shifted peaks, suggesting a more electron-rich environment at the metal interface. The suppression of FeOOH vibrational signatures in the CIP@SiO_2_ sample reinforces the role of the SiO_2_ shell as a kinetic barrier to oxidation. Additionally, the presence of D (∼1360 cm^−1^) and G (∼1570 cm^−1^) bands, typically associated with disordered (sp^3^) and graphitic (sp^2^) carbon structures, respectively,^41^ indicates residual organic moieties from the sol-gel process. Furthermore, CIP@γ-FeOOH exhibits a clear oxygen vacancy (O_v_) signal at a g-factor (g) = 2.003 in the EPR spectrum (Figure 2D), indicating the presence of O_v_.

**Figure 3:**
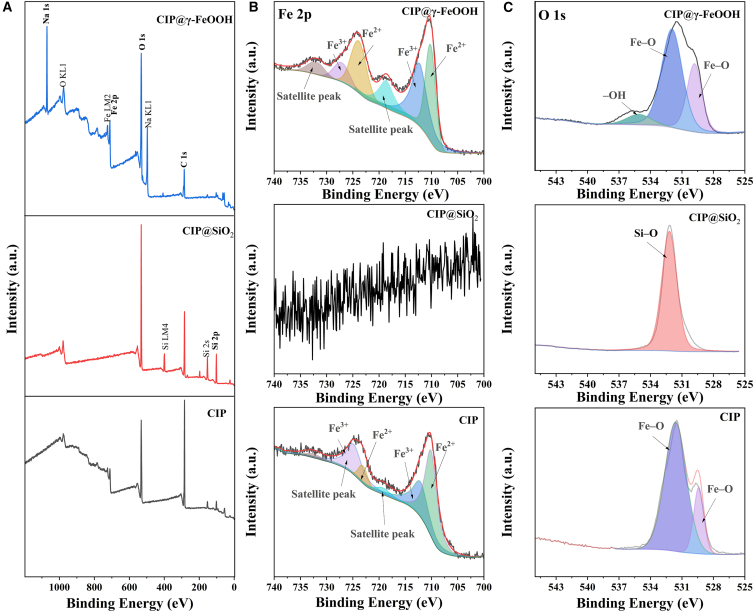
XPS characterization of CIP-based materials XPS spectra of CIP, CIP@SiO_2_, and CIP@γ-FeOOH: (A) survey spectrum, (B) Fe 2p, and (C) O 1s.

Figure 3A shows the full XPS patterns of CIP, CIP@SiO_2_, and CIP@γ-FeOOH. In CIP@SiO_2_, a prominent Si 2p peak emerges at 103.5 eV, corresponding to non-stoichiometric SiO_1.8_ species,^42^ while the Na 1s peak at 1071.2 eV in CIP@γ-FeOOH reflects residual sodium ions introduced during the alkali treatment. These peaks serve as fingerprints of the modified surface chemistry. High-resolution Fe 2p spectra (Figure 3B) provide further insight into iron oxidation states. Pristine CIP shows an Fe^2+^ peak at 710.5 eV and 723.6 eV, and a Fe^3+^ doublet at 712.5 eV and 725.6 eV,^43^^,^^44^ confirming a Fe^2+^ and Fe^3+^-rich surface. After SiO_2_ encapsulation, no Fe^2+^/Fe^3+^ signals are observed, which is likely because the thickness of the SiO_2_ shell exceeds the detection depth of XPS. In CIP@γ-FeOOH, the Fe^2+^ peak shifts slightly to 710.6/723.7 eV, while the Fe^3+^ doublet becomes more prominent (at 712.8/727.3 eV), suggesting deep oxidation into higher-valence iron species promoted by the γ-FeOOH shell.^45^^,^^46^ Meanwhile, the enhanced intensity of the Fe 2p satellite peak typically indicates an increase in the Fe oxidation state (Fe^2+^ → Fe^3+^), reflecting a higher oxidation degree, more pronounced charge-transfer effects, and an intensified surface or interfacial oxidation environment (the Fe^2+^/Fe^3+^ ratio, see more details in Supporting Information S2). The O 1s spectra (Figure 3C) further support these observations. In CIP, a broad Fe–O signal appears at 529.3 eV and 531.9 eV.^47^ In CIP@SiO_2_, a lattice oxygen peak at 532.2 eV corresponds to Si–O, indicating an efficient SiO_2_ coating on the CIP surface. In CIP@γ-FeOOH, lattice Fe–O components are observed at 529.8 eV and 531.9 eV. Additionally, a weak hydroxyl signal at 535.1 eV confirms the presence of adsorbed –OH species, which is consistent with the FTIR band observed at 1427 cm^−1^ (Figure 2B).

**Figure 4:**
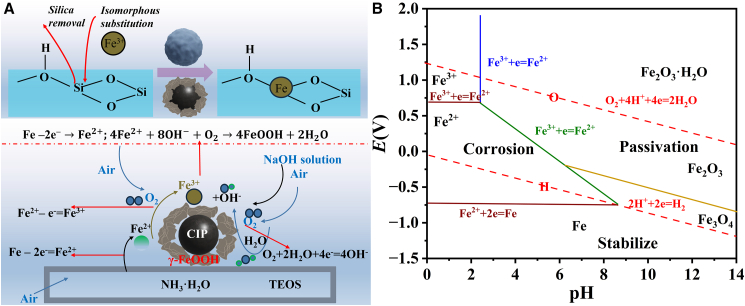
Proposed formation mechanism and thermodynamic analysis for CIP@γ-FeOOH (A) Schematic illustration of the growth mechanism of γ-FeOOH on CIP surfaces. (B) Pourbaix diagram of Fe-based systems.

As illustrated in Figure 4A, the formation of γ-FeOOH nanosheets on CIP proceeds via a multistep chemical evolution process under controlled alkaline hydrothermal conditions. The process begins with surface activation mediated by O_v_ and hydroxyl groups, whose generation has been confirmed by EPR、 FTIR, and XPS analyses. These reactive sites play a pivotal role in initiating subsequent phase transformation and nanostructure assembly. Under mildly alkaline conditions (pH ≈ 9), TEOS undergoes hydrolysis to form an amorphous SiO_2_ shell on the CIP surface, providing initial passivation and templating for interfacial reactions (Equation 1). Simultaneously, spontaneous oxidation of surface Fe atoms occurs, producing Fe^2+^ ions that partially enrich the interface (Equation 2). During subsequent alkaline hydrothermal treatment (1.5 mol L^−1^ NaOH, 60 °C), the SiO_2_ shell gradually dissolves, leading to the release of SiO_2_ species and exposure of the underlying Fe surface (Equation 3). This exposure triggers further oxidation of Fe^2+^ to Fe^3+^ (Equation 4), while the elevated pH and dissolved oxygen drive co-precipitation reactions involving Fe^2+^, OH^−^, and O_2_ to yield γ-FeOOH nuclei (Equation 5). The resulting FeOOH complexes are electrostatically attracted to the hydroxylated CIP surface, where they undergo site-selective nucleation and growth. As etching proceeds, the γ-FeOOH nanocrystals assemble into lamellar networks through oriented attachment and evolve into flower-like superstructures via Ostwald ripening.^48^ The observed transition from nanosheet aggregates to well-defined hierarchical morphologies (Figures 2F–2I) corresponds to growth stages at 8, 16, 24, and 48 h, respectively.

(Equation 1) Si(OCH_2_CH_3_)_4_ + 2H_2_O → SiO_2_ + 4C_2_H_5_OH

(Equation 2) Fe – 2e^–^ → Fe^2+^

(Equation 3) SiO_2_ + 2NaOH → Na_2_SiO_3_ + H_2_O

(Equation 4) Fe^2+^ – e^–^ → Fe^3+^

(Equation 5) 4Fe^2+^ + 8OH^−^ +O_2_ → 4FeOOH + 2H_2_O

According to the Fe–H_2_O pH–potential diagram^49^ (Figure 4B), the reaction environment (pH > 9, ambient redox potential) thermodynamically favors the formation of γ-FeOOH over other iron oxyhydroxide polymorphs. The preferential emergence of the γ-phase is further supported by the absence of α-FeOOH vibrational signatures in Raman spectra. Collectively, these results confirm that the surface redox microenvironment—modulated by SiO_2_ etching, pH elevation, and dissolved oxygen—is the key determinant in achieving phase-selective and morphology-controlled γ-FeOOH growth.

### Ferromagnetic/antiferromagnetic interface pinning enhanced electromagnetic wave absorption performance

In the field of EMW absorption performance research, *RL* and impedance matching, as two critical parameters, play a decisive role in achieving excellent EMW performance. According to the transmission line theory, the relevant calculation formulas^1^^,^^4^^,^^50^ are expressed as follows:

(Equation 6) RL=20lg|(Z-1)/(Z+1)|

(Equation 7) $$Z=\frac{Z_{\text{in}}}{Z_{0}}=\sqrt{\frac{\mu_{r}}{\varepsilon_{r}}}\tanh\left(j\frac{2\pi fd}{c}\sqrt{\mu_{r}\varepsilon_{r}}\right)$$

Here, *Z* represents the normalized input impedance relative to free space, *Z*_in_ is the input impedance of the material, *Z*_0_ is the characteristic impedance of free space, and *d* is the absorber thickness.

**Figure 5:**
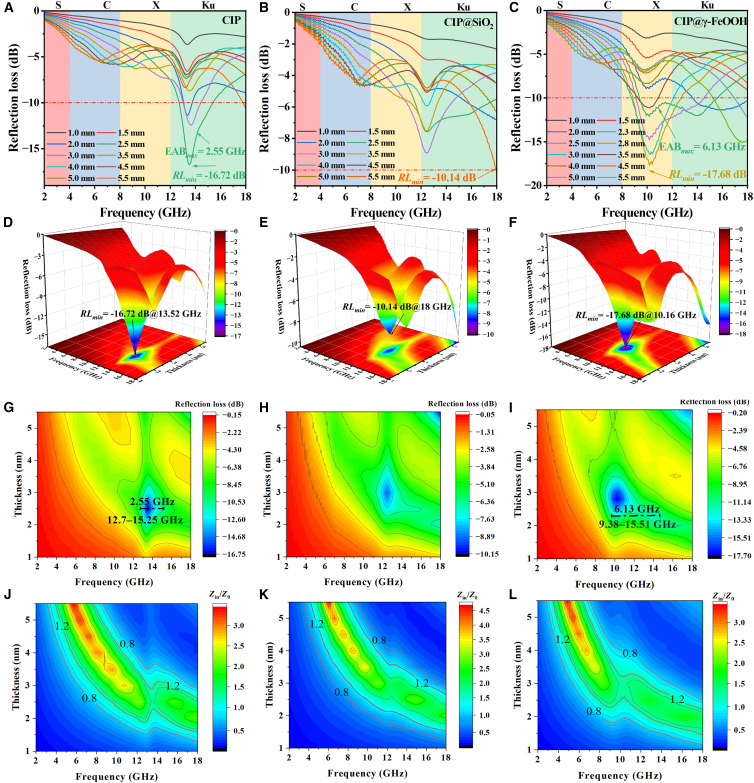
Two-dimensional, three-dimensional *RL* graphs and impedance matching plots of materials (A, D, G, and J) CIP, (B, E, H, and K) CIP@SiO_2_ and (C, F, I, and L) CIP@γ-FeOOH.

The two-dimensional (2D) and the three-dimensional (3D) *RL* graphs of CIP, CIP@SiO_2,_ and CIP@γ-FeOOH are shown in Figures 5A–5I). For pristine CIP (Figure 8A), the *RL*_min_ is −16.72 dB at 13.76 GHz (Ku band), showing poor EMW absorption performance and a relatively high absorption *f*. The EAB_max_ is only 2.55 GHz (12.7–15.25 GHz). After SiO_2_ coating (Figure 8B), the EMW absorption ability is further weakened, and no obvious EAB_max_ is observed due to the reduced magnetic contribution. In contrast, CIP@γ-FeOOH exhibits markedly enhanced absorption properties. At a thickness of 2.8 mm, an *RL*_min_ of −17.68 dB is obtained at 10.16 GHz (X band). Furthermore, at an optimized thickness of 2.3 mm, an EAB_max_ of 6.13 GHz (9.38–15.51 GHz) is achieved, representing a 140% increase relative to pristine CIP and covering most of the X and Ku bands. This enhancement is likely attributed to the nanosheet-like surface, which provides additional dissipation pathways. Effective EM absorption requires both strong attenuation and good impedance matching, which ensures maximum energy transfer from the incident wave to the absorber. When |*Z*_in_/*Z*_0_| = 0.8–1.2, optimal impedance matching is achieved. Compared to CIP, which shows limited impedance matching in the Ku band, CIP@γ-FeOOH displays a significantly expanded impedance matching region in the X band. Without the SiO_2_ transition layer, the impedance matching is significantly worse, and the material shows almost no EMW absorption performance, as discussed in Supporting Information S4. The *RL* curve of CIP@γ-FeOOH exhibits larger fluctuations at lower *f* compared to the other two materials, which may be due to magnetic resonance arising from the anisotropic, sheet-like structure of CIP@γ-FeOOH, introducing enhanced magnetic-dipole resonances and domain-wall or natural resonance loss mechanisms, along with dielectric loss components reflecting multiple polarization and relaxation mechanisms.

To better position the performance of CIP@γ-FeOOH, we compared it with several representative CIP-based composites reported in recent years, as summarized in Table 1. In contrast, although our CIP@γ-FeOOH adopts a simpler binary architecture and a lower filler content (60 wt. %), it still delivers a competitive EAB_max_ of 6.13 GHz and improved low-*f* attenuation.

**Table 1:** Comparison of the EMW absorption performance of different CIP-based coating samples

Composite	Ratio (wt. %)	*RL*_min_ (dB) @ *f* (GHz)	*d* (mm)	EAB_max_ (GHz)	*d* (mm)	Reference
CIP	80	–27.57@9.6	2.0	5.49(7.94–13.43)	2.0	Chen et al.^11^
CIP@SiO_2_@ Mn_0.__6_Zn_0.__4_Fe_2_O_4_	80	–44.24@11.57	2.0	7.12(9.04–16.16)	2.0	Chen et al.^11^
CIP@SiO_2_ @Carbon	80	–62.2@11.9	1.64	8.6(9.4–18)	1.64	Li et al.^15^
CIP@TiO_2_	80	–46.07@5.04	3.1	7.76(9.36–17.12)	1.5	Su et al.^16^
CIP/PDA@MWCNTs	20	–30.69@9.68	1.5	9.68(6.48–16.16)	1.5	Zheng et al.^18^
CIP	60	–16.72@13.52	2.5	2.55(12.7–15.25)	2.5	This work
CIP@γ-FeOOH	60	–17.68@10.16	2.8	6.13(9.38–15.51)	2.3	This work

**Figure 6:**
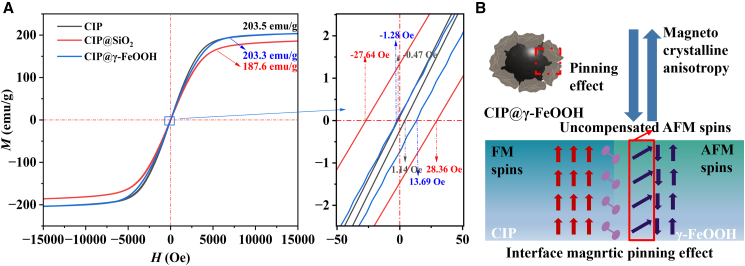
Magnetic properties of CIP-based materials (A) Hysteresis loops of CIP, CIP@SiO_2_, and CIP@γ-FeOOH. (B) Exchange bias behavior of CIP@γ-FeOOH.

**Figure 7:**
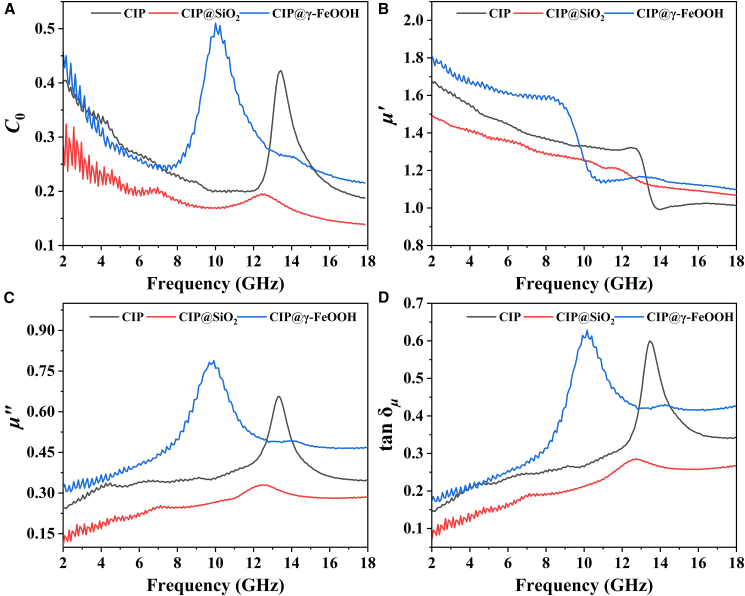
Magnetic properties of CIP-based materials (A) Magnetic loss factor (*C*_0_), (B) *μ*′, (C) *μ*″, and (D) tan δ_*μ*_ of the CIP, CIP@SiO_2_ and CIP@γ-FeOOH.

**Figure 8:**
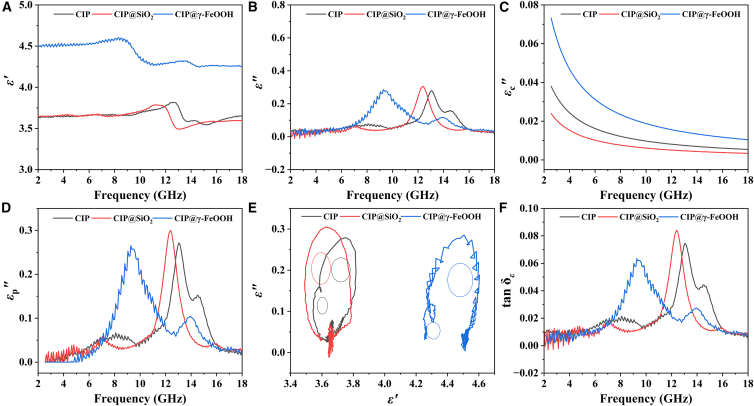
Dielectric properties of CIP-based materials (A) *ε*′, (B) *ε*″, (C) *ε*_c_″, (D) *ε*_p_″, (E) Cole-Cole plot, and (F) tan δ_*ε*_ of CIP, CIP@SiO_2_, and CIP@γ-FeOOH.

Figures 6, 7, and 8 show the EM performance of CIP, CIP@SiO_2_, and CIP@γ-FeOOH, including hysteresis loops, magnetic loss factor (*C*_0_), real and imaginary parts of complex permeability (*μ*′, *μ*″) and permittivity (*ε*′, *ε*″), magnetic and dielectric loss tangents (tan δ_*μ*_, tan δ_*ε*_). Figure 6A shows that the pristine CIP exhibits typical soft magnetic properties with a high saturation magnetization (*M*_*s*_) of 203.5 emu/g. After encapsulation with SiO_2_ (CIP@SiO_2_), *M*_s_ decreases to 187.6 emu/g, corresponding to a 7.8% reduction. This decline arises from two combined effects: First, the dielectric SiO_2_ shell increases interfacial impedance, enhancing reflection and reducing the effective EM penetration depth; Second, the introduction of a nonmagnetic shell causes volumetric dilution of the magnetic phase. Following hydrothermal oxidation, CIP@γ-FeOOH restores *M*_s_ to 203.3 emu/g, nearly identical to that of the pristine material. This recovery is attributed to a magnetic compensation mechanism in which the weakly ferromagnetic γ-FeOOH shell interacts with the core, reorienting interfacial magnetic moments and forming a more effective exchange-coupled network. A higher *M*_s_ indicates a stronger magnetic response and the ability to effectively absorb EMW over a wider *f* band. The magnetic hysteresis behavior shown in Figure 6A, together with the exchange bias analysis in Figure 6B, provides further insight into the interfacial magnetic coupling within the composite structures. Among the three materials, CIP@γ-FeOOH exhibits a notably asymmetric hysteresis loop, with distinct left and right coercive fields measured at −1.28 Oe and 13.69 Oe, respectively. This asymmetric shift confirms the presence of exchange bias, a phenomenon not observed in either pristine CIP or CIP@SiO_2_. Although pristine CIP contains a weak γ-FeOOH component (Figures 3B and 3C), its amount is too low, and the surface distribution is highly discontinuous, preventing the formation of a coherent FM-AFM interface. Furthermore, the *H*_c_ reflects magnetic anisotropy, and a slight increase in *H*_c_, facilitated by FM/AFM interfacial coupling, broadens the relaxation spectrum, enabling multiple magnetic-loss mechanisms.

The magnetic loss behavior of CIP can be decomposed into four primary mechanisms, including domain wall resonance, hysteresis loss, eddy current loss, and natural resonance. In this study, the contribution from domain wall resonance is negligible, as its characteristic frequency is typically below 100 MHz, which is far lower than the experimental range of 2–18 GHz.^51^ The magnetic hysteresis loops in Figure 7A exhibit narrow-waisted profiles across all samples, suggesting that hysteresis loss is not a major contributor. As a result, magnetic loss is mainly governed by the combined effects of eddy current and natural resonance. To evaluate their respective contributions, the magnetic loss factor *C*_0_ is employed, defined as *C*_0_ = *μ″*(*μ′*)^−2^*f*^-1^.^52^ The experimental data reveal *f*-dependent peaks across 12–16 GHz for CIP, 10–14 GHz for CIP@SiO_2_, and 8–12 GHz for CIP@γ-FeOOH. These variations indicate that strong natural resonance dominates within these *f* bands, whereas eddy current loss prevails outside them.^53^ Because CIP@γ-FeOOH contains FM/AFM interfaces formed by two magnetic components, exchange resonance also contributes to the resonance peaks observed in the X band. The corresponding evolution of *μ′* and *μ″* are depicted in Figures 7B and 7C). Both *μ′* and *μ″* are notably diminished in CIP@SiO_2_ due to the combined influence of magnetic dilution and the spatial isolation imposed by the SiO_2_ shell. In contrast, CIP@γ-FeOOH exhibits markedly enhanced *μ″* over the 2–12 GHz range. This enhancement originates from interfacial magnetic pinning,^54^^,^^55^ where exchange coupling between the CIP core and the γ-FeOOH shell produces a unidirectional anisotropy field. According to Snoek’s limit^56^ $\left(f_{r}\left(\mu_{r}-1\right)=\frac{\gamma }{2\pi }4\pi M_{s}\right)$, materials with similar *M*_s_ values (Figure 6A) generally exhibit a trade-off between *μ*_*r*_ and resonance *f*. The observed increase in low-*f μ*′/*μ″* of CIP@γ-FeOOH suggests that interfacial coupling effectively extends the Snoek limit, enabling broader magnetic resonance and enhanced low-*f* magnetic responsiveness. For CIP@γ-FeOOH, the tan *δ*_*μ*_ (Figure 7D) also exhibits absorption peaks at the same positions as *μ*″ (Figure 7C), which coincide with the *RL*_min_ peaks in Figure 5C, thereby confirming that magnetic loss is the dominant EMW dissipation mechanism.

As shown in Figures 8A and 8B, CIP@γ-FeOOH exhibits a substantially enhanced dielectric response in the low-*f* region. This enhancement is primarily attributed to the flower-like surface architecture, which increases *ε*′ through Maxwell-Wagner interfacial polarization.^57^ The effect arises from interfacial charge accumulation between the FM CIP core and the AFM γ-FeOOH shell, which collectively amplifies the polarization intensity. The shift of the *ε*″ peak toward lower *f*, together with its suppression at higher *f*, indicates a damped dipolar relaxation process. Under a varying electromagnetic field, the electric potential dynamically reorients, resulting in polarization relaxation that enhances EMW absorption by converting electromagnetic energy into Joule heat.^58^ This process effectively reduces reflection and secondary interference. To better understand the dielectric loss mechanisms, we separated the imaginary part of *ε*″ into conductive loss (*ε*″_c_) and polarization loss (*ε*″_p_) using the Debye relaxation model^59^ (details provided in Supporting Information S4). As shown in Figure 8C, CIP@γ-FeOOH exhibits the highest conductive loss, which can be attributed to the conductive FeO_*x*_ phases and the Fe^2+^↔Fe^3+^ redox transitions, which further lower the energy barrier for electron transitions. Additionally, Figure 8D demonstrates that CIP@γ-FeOOH shows the most pronounced polarization loss between 8 and 12 GHz, which aligns well with the *RL*_min_ region (Figure 5C). Comparing Figures 8C and 8D, it is evident that the dielectric loss in CIP@γ-FeOOH is primarily driven by interfacial polarization. To gain further insight into the relaxation behavior, the Cole-Cole plot (*ε*″ vs. *ε*′) was analyzed based on the Debye relaxation theory. The equation used for fitting is

(Equation 8) $${\left(\varepsilon^{\prime }-\frac{\varepsilon_{s}+\varepsilon_{\infty }}{2}\right)}^{2}+{\left(\varepsilon^{\prime \prime }\;\right)}^{2}={\left(\frac{\varepsilon_{s}-\varepsilon_{\infty }}{2}\right)}^{2}$$

where *ε*_s_ and *ε*_∞_ represent the static and high-*f* dielectric constants, respectively. Ideal Debye relaxation should yield a single semicircle; however, deviations from this ideal indicate the presence of multiple relaxation processes. As shown in Figure 8F, CIP, CIP@SiO_2_, and CIP@γ-FeOOH exhibit 2, 1, and 2 semicircles, respectively, suggesting progressively more complex relaxation behaviors. The more distorted Cole-Cole curve of CIP@γ-FeOOH implies the coexistence of multiple polarization processes, as surface dipoles and abundant interfacial regions introduce diverse polarization-relaxation pathways. This further confirms the intricate and highly coupled polarization network within CIP@γ-FeOOH.

Moreover, the tan δ_*ε*_ (Figure 8D) shows a resonance peak at the same positions as *ε*″ (Figure 8B), but slightly shifted toward lower *f* compared with the magnetic loss (Figure 7D) and the *RL*_min_ peaks (Figure 5C), and the order of magnitude of dielectric loss is significantly lower than that of magnetic loss. These results confirm that magnetic loss is the predominant attenuation mechanism. In addition, CIP@γ-FeOOH reaches its maximum *α* in the X-band, corresponding to its strongest EMW absorption capability. Further details are provided in Supporting Information S5.

**Figure 9:**
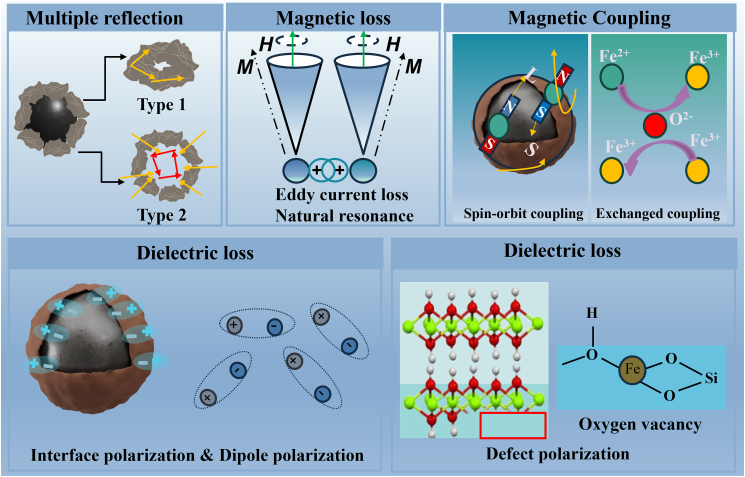
Proposed EMW absorption mechanism of CIP@γ-FeOOH composite

As illustrated in Figure 9, the EMW dissipation mechanisms of CIP@γ-FeOOH mainly include multiple reflections, magnetic loss, and dielectric loss. The hierarchical core-shell and flower-like architectures introduce internal air voids and heterogeneous interfaces, which facilitate deeper wave penetration and enhanced energy dissipation within the absorber matrix. The flower-like γ-FeOOH shell consists of densely stacked and angular nanosheets that produce irregular interfaces, gaps, edges, and local dielectric discontinuities around the spherical CIP core. These features generate multiple scattering centers. Magnetic loss dominates the conversion of EM energy, governed primarily by natural resonance and eddy current effects. The presence of exchange bias fields (Figure 6A) further modulates the magnetic anisotropy landscape, enhancing spin-orbit and exchange coupling,^60^ thereby broadening the resonance bandwidth and improving the low-*f* permeability. Dielectric loss originates from multiple polarization processes. Surface functional groups induce Debye-type dipolar relaxation, while interfacial polarization arises from accumulated charges at the core-shell boundary. In addition, O_v_ introduced during etching acts as an electron trap, promoting hopping conduction and defect-related polarization. These defects distort the Fe–O coordination environment, generate fluctuating local dipoles, and promote carrier hopping between vacancy-associated states, all of which contribute to increased dielectric loss. These magnetic and dielectric loss mechanisms are tightly coupled, forming synergistic dissipation pathways that enable broadband and efficient microwave absorption performance.

In summary, this work presents a transition-layer-guided strategy for constructing CIP@γ-FeOOH heterostructures, achieving integrated control over interfacial structure and EM behavior, and breaking the key scientific issue of narrow EAB under low filling conditions in CIP. The introduction of a sacrificial SiO_2_ shell enables selective etching and *in-situ* oxidation, leading to the formation of a defect-rich γ-FeOOH shell. This architecture establishes effective exchange coupling at the FM/AFM interface, where the resulting interfacial magnetic pinning broadens the resonance bandwidth and enhances low-*f* magnetic responsiveness, thereby improving impedance matching and promoting EMW absorption. The elucidation of particle growth mechanisms and polarization loss pathways provides fundamental insights into multiscale interfacial interactions and energy dissipation behavior. These findings offer a new perspective for interface engineering in iron-based microwave absorbers and advance the rational design of next-generation EMW attenuation materials.

### Limitations of the study

This study constructed hierarchical CIP@γ-FeOOH core-shell heterostructures through a transition-layer-guided oxidation strategy. The precise influence of the antiferromagnetic shell thickness on the exchange bias mechanism requires further in-depth investigation. Additionally, the feasibility of industrial-scale production and the potential for multifunctional applications have not yet been explored. Despite the above-mentioned limitations, the “transition-layer-guided oxidation” strategy provides a new idea for the structural design and interfacial engineering of high-performance electromagnetic wave absorbing materials.

## Resource availability

### Lead contact

Further information and requests for resources and reagents should be directed to and will be fulfilled by the lead contact, Miao Jiang (jiangmiao@bit.edu.cn).

### Materials availability

This study did not generate new unique reagents.

### Data and code availability

• Data: Data reported in this article will be shared by the lead contact upon request.
• Code: This article does not report original code.
• Additional information: Any additional information required to reanalyze the data reported in this article is available from the lead contact upon request.

## Acknowledgments

This work was partly supported by the 10.13039/501100001809National Natural Science Foundation of China (Grant No. 12204036, 52471252), the National Key Laboratory Foundation of Science and Technology on Materials under Shock and Impact (6142902230101), and the 10.13039/501100021171Guangdong Basic and Applied Basic Research Foundation (2025A1515011206). The authors would like to thank Analytical & Testing Center of 10.13039/501100005085Beijing Institute of Technology, 10.13039/501100002726Beijing Normal University, and Scientific Compass www.shiyanjia.com for XRD, SEM, TEM, VSM and XPS tests performed in this work.

## Author contributions

Y.L.: conceptualization, methodology, investigation, data curation, and writing; L.L.: resources and software; H.Z.: formal analysis and conceptualization; L.G.: resources and supervision; Z.M.: methodology and supervision; Q.C.: supervision, project administration, and review; M.J. (Corresponding author): supervision, funding acquisition, editing, and project administration. All authors have given approval to the final version of the article.

## Declaration of interests

The authors declare that they have no known competing financial interests or personal relationships that could have appeared to influence the work reported in this article.

## STAR★Methods

### Key resources table

**Table:** 

REAGENT or RESOURCE	SOURCE	IDENTIFIER
**Chemicals, peptides, and recombinant proteins**		

CIP	BASF	EW, D50: 3–4 μm, Purity: 99.9% https://electronics-electric.basf.com/
Tetraethyl orthosilicate	Aladdin	CAS:78-10-4, MV:208.33 https://www.aladdin-e.com/zh_cn/t110595.html
NH_3_·H_2_O	Macklin	CAS:1336-21-6, MV:35.05 https://www.macklin.cn/products/A834475
NaOH	Aladdin	CAS:1310-73-2, Purity: 98% https://www.aladdin-e.com/zh_cn/s580606.html

**Software and algorithms**		

Origin 2025	OriginLab	https://www.originlab.com
MDI Jade 6	Materials Data	https://www.materialsdata.com/prodjd.html
OMNIC	Thermo Fisher Scientific	https://www.thermofisher.cn/cn/zh
Avantage	Thermo Fisher Scientific	https://www.thermofisher.cn/cn/zh
CST Studio Suite 2024	Dassault Systèmes	https://www.3ds.com

### Experimental model and study participant details

Omitted, as our study does not involve biological models.

### Method details

#### Materials

##### Preparation of CIP@γ-FeOOH

Specifically, 1 g of CIP was first dispersed in a mixture of 200 mL of anhydrous ethanol and 15 mL of deionized water, followed by stirring for 2 h. Subsequently, a pre-prepared solution of 1 mL of TEOS in 100 mL of anhydrous ethanol was added dropwise via a constant-pressure dropping funnel. Thereafter, 20 mL of ammonia solution was added to initiate the sol-gel reaction. The mixture was continuously stirred at 40°C for 8 h. The resulting product was magnetically separated and dried at 80°C. The dried powder was then immersed in 200 mL of 1.5 mol/L sodium hydroxide solution and subjected to mechanical stirring at 60°C for 48 h. During this period, 10 cycles of intermittent sonication (20 min per cycle) were applied at regular intervals to promote selective etching. The final product was magnetically recovered, thoroughly rinsed with deionized water, and dried in a convection oven at 60°C for 12 h.

#### Characterization

Morphological features were examined using field-emission scanning electron microscopy (SEM, Hitachi S-4800) and transmission electron microscopy (TEM, FEI Tecnai TF20). The phase structure was analyzed using X-ray diffraction (XRD, Cu K*α*_1_ radiation, *λ* = 1.54056 Å), with a 2*θ* scanning range of 5°–90° at a rate of 20°/min. Fourier transform infrared spectra (FTIR, 400–4000 cm^-1^) were recorded on a Bruker Vector33 spectrometer. Oxygen vacancies were identified by electron paramagnetic resonance (EPR, Bruker EMX-plus) spectroscopy spectrometer. Surface chemical states were determined by X-ray photoelectron spectroscopy (XPS, Thermo Scientific K-Alpha) with Al K*α* excitation. Magnetic performance was characterized at room temperature using a vibrating sample magnetometer (VSM, LakeShore 7404). EM parameters in the frequency range of 2–18 GHz were measured using a vector network analyzer (VNA, Agilent E5071C) via the coaxial line method. For this purpose, CIP-based powders were homogeneously mixed with paraffin wax (mass ratio of 3:2) and pressed into toroidal rings (inner diameter 3.04 mm; outer diameter 6.95 mm; thickness 2.00 mm).

### Quantification and statistical analysis

Graphs in the main text and Supporting Information were generated from the raw data using Origin 2025. XRD patterns were analyzed using MDI Jade 6. FTIR spectra were processed using OMNIC. XPS data were analyzed using Thermo Scientific Avantage. Electromagnetic simulations were performed using CST Studio Suite 2024.
