# Supplementary material for: Enhanced electromagnetic wave absorption performance by introducing exchange bias in a CIP@γ-FeOOH heterostructure
Source: iScience. 2026 Jan 29;29(2):114822. doi: 10.1016/j.isci.2026.114822 (PMC12915284; doi:10.1016/j.isci.2026.114822)
Supplement: Document S1. Figures S1–S3 and Table S1 [file mmc1.pdf]

iScience, Volume *29*

Supplemental information

Enhanced electromagnetic wave absorption performance by introducing exchange bias in a CIP@γ-FeOOH heterostructure

Yunpeng Li, Luyang Li, Haojie Zhang, Jixing Bai, Lihong Gao, Zhuang Ma, Qi Cao, and Miao Jiang

This PDF includes:

- ☐ **Section S1:** The surface oxidation morphology of CIP in alkaline solution.
- ☐ **Figure S1.** (a) CIP surface oxidation morphology diagram without SiO_2 buffer layer in alkaline solution (a) low multiple (b) high multiple.
- ☐ **Section S2:** The ratio of Fe^{2+} to Fe^{3+} in CIP and CIP@γ -FeOOH.
- ☐ **Table S1.** Peak separation description of Fe 2p.
- ☐ **Section S3:** Electromagnetic performance of CIP oxidation.
- ☐ **Figure S2.** Electromagnetic performance of CIP oxidation. (a) reflection loss; (b) impedance matching.
- ☐ **Section S4:** Separation of Dielectric Loss Components Based on Debye Relaxation Theory.
- ☐ **Section S5:** The attenuation capacity of the sample.
- ☐ **Figure S3.** Attenuation constant (α) of CIP, CIP@SiO_2, CIP@γ-FeOOH.

Section S1: The surface oxidation morphology of CIP.

The SiO_2 transition layer provides chemical inertness and a conformal interface, preventing rapid and uncontrolled corrosion of the CIP surface during hydrothermal oxidation. Without this protective layer, CIP undergoes direct, aggressive surface oxidation, leading to irregular, rough, and discontinuous FeOOH deposits instead of a uniform shell, as shown in Figure S1.

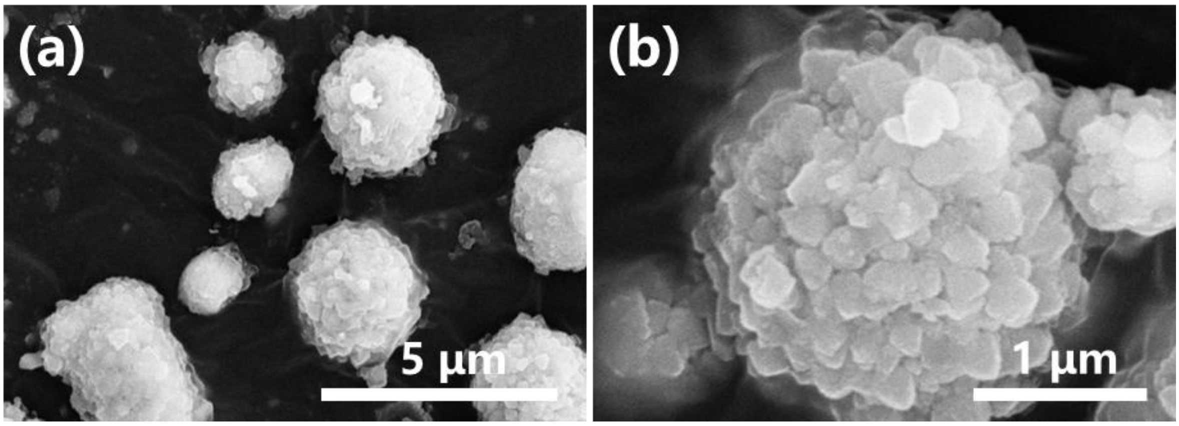

Figure S1. (a) CIP surface oxidation morphology diagram without SiO_2 buffer layer in alkaline solution (a) low multiple (b) high multiple.

Section S2: The ratio of Fe^{2+} to Fe^{3+} in CIP and CIP@γ -FeOOH.

Table S1. Peak separation description of Fe 2p.

Sample	Peak	Peak position	Height	Height ratio	Area	Area ratio	FWHM
CIP	Fe $2p_{3/2}$ Fe^{3+}	712.5	3182.37	0.27	10345.87	0.23	3.12
	Fe $2p_{3/2}$ Fe^{2+}	710.5	11929.72	1	44998.81	1	3.62
	Fe $2p_{1/2}$ Fe^{3+}	725.6	4650.56	0.39	7753.32	0.17	1.6
	Fe $2p_{1/2}$ Fe^{2+}	723.6	4300.64	0.36	5287.83	0.12	1.18
CIP@γ-FeOOH	Fe $2p_{3/2}$ Fe^{3+}	712.8	11665.36	0.44	60046.43	0.94	4.94
	Fe $2p_{3/2}$ Fe^{2+}	710.6	26547.86	1	64177.05	1	2.32
	Fe $2p_{1/2}$ Fe^{3+}	727.3	5790.9	0.22	25222.31	0.39	4.18
	Fe $2p_{1/2}$ Fe^{2+}	723.7	12816.62	0.48	35390.09	0.55	2.65

From Table S1, both the peak height and peak area of Fe^{2+} and Fe^{3+} in CIP@γ-FeOOH increase markedly, and the Fe^{2+}/Fe^{3+} area ratio decreases from 2.8 to 1.17. This indicates a substantial enhancement in the surface oxidation state after modification.

Section S3: Electromagnetic performance of CIP oxidation.

The absorption performance and impedance matching of CIP subjected to direct oxidation without a SiO_2 buffering layer are shown in Figure S2. As illustrated, at a thickness of 5.5 mm, only a narrow impedance-matching region appears in the high-*f* range, yielding an EAB_{max} of merely 1.35 GHz. This result indicates that direct oxidation significantly deteriorates the microwave absorption capability of CIP.

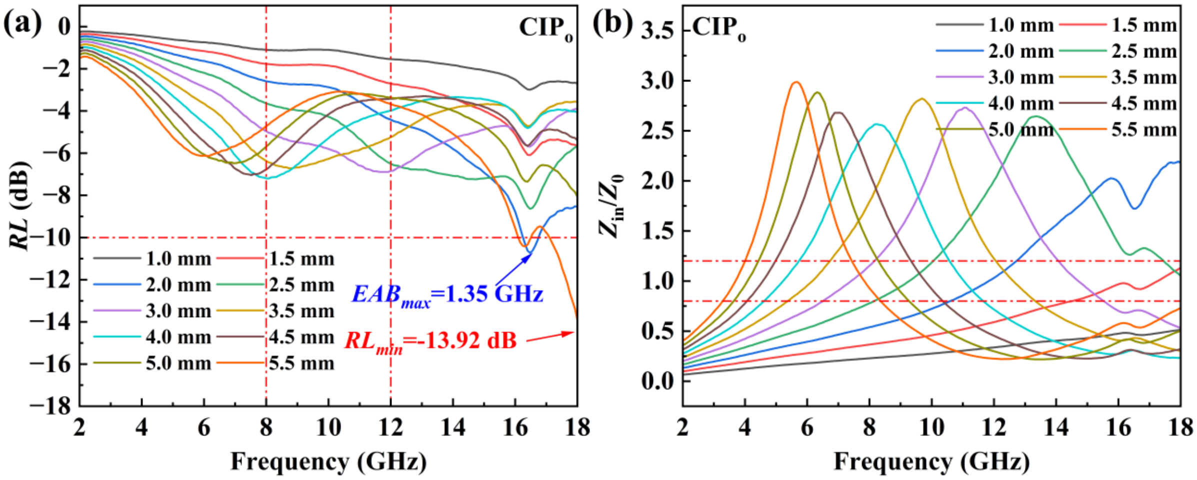

Figure S2. Electromagnetic performance of CIP oxidation. (a) reflection loss; (b) impedance matching.

Section S4: Separation of Dielectric Loss Components Based on Debye Relaxation Theory

Understanding the EMW absorption mechanism requires a detailed analysis of dielectric loss behavior. According to Debye relaxation theory [1], the imaginary part of the complex permittivity (ε'') consists of two main contributions: conduction loss (ε_c'') and polarization loss (ε_p''). ε_c'' originates from the motion of free carriers under an electric field and dominates in the low-frequency region. ε_p'' arises from dipole relaxation, interfacial polarization, and other polarization effects, typically observed over a broader frequency range. The formula is as follows [2,3]:

$$\varepsilon''=\varepsilon_c''+\varepsilon_p'' \quad \text{(Equation 1)}$$

$$\varepsilon_c''=\frac{\sigma}{\omega\varepsilon_0} \quad \text{(Equation 2)}$$

$$\varepsilon_p''=\frac{(\varepsilon_s-\varepsilon_\infty)\omega\tau}{1+(\omega\tau)^2} \quad \text{(Equation 3)}$$

where ε_s is the static permittivity, ε_∞ is the high-frequency limit permittivity, $\omega = 2\pi f$ is the angular frequency, τ is the relaxation time, σ is the electrical conductivity, and ε_0 is the vacuum permittivity (8.85×10^{-12} F·m^{-1}). In the low f region, ε_c'' dominates due to the increasing contribution of the $\sigma / (\omega\varepsilon_0)$ term, which leads to a characteristic inverse relationship between ε'' and f. Assuming a constant electrical conductivity, ε'' is approximately proportional to $1/f$, enabling the extraction of the conduction loss component via linear fitting. The polarization loss can then be obtained by subtracting ε_c'' from the total ε''. In the high-f region, ε_c'' becomes negligible, and ε'' is primarily governed by dipolar relaxation processes, often manifested as f-dependent peaks in ε'' and dielectric loss tangent (tan δ_ε). These relaxation behaviors can also be visualized using Cole–Cole plots (ε'' vs ε'), where each semicircle corresponds to an individual relaxation process. The presence of multiple semicircles indicates multiple dipolar or interfacial polarization mechanisms contributing to dielectric loss.

Section S5: The attenuation capacity of the sample.

EMW absorption is fundamentally determined by the material's ability to dissipate energy, wherein incident EMW is transformed into thermal energy through dielectric and magnetic losses. A key parameter that governs this dissipation is the attenuation constant α, which characterizes how rapidly the wave amplitude decreases within the medium. Physically, this parameter correlates with multiple internal reflections and extended propagation paths. The α is given by [4]:

$$\alpha = [((2)^{1/2}\pi f)/c] / \{(\mu''\varepsilon''-\mu'\varepsilon') + [(\mu''\varepsilon''-\mu'\varepsilon')^2 + (\varepsilon'\mu''- \varepsilon''\mu')^2]^{1/2}\}^{1/2} \quad \text{(Equation 4)}$$

where c is the speed of light in vacuum. As shown in Figure 7(h), the α of the three materials differ significantly. CIP@SiO_2 exhibits the lowest overall attenuation. In the 13–15 GHz range, CIP shows higher α than CIP@γ-FeOOH, but outside this interval, CIP@γ-FeOOH demonstrates superior attenuation performance.

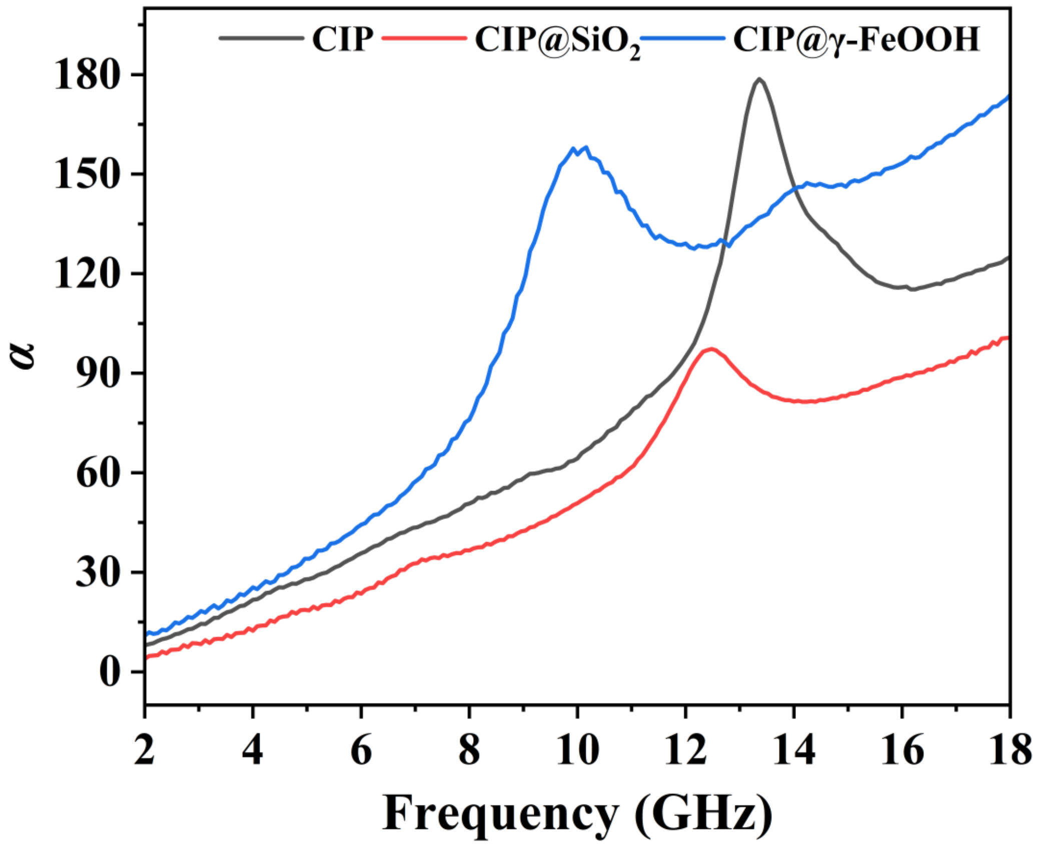

Figure S3. Attenuation constant (α) of CIP, CIP@SiO_2, CIP@γ-FeOOH.

Supplementary References

1. Kremer, F., Kipnusu, W.K., and Fränzl, M. (2022). Orientation polarization spectroscopy—toward an atomistic understanding of dielectric relaxation processes. Int. J. Mol. Sci. *23*, 8254.
2. Qin, M., Zhang, L., and Wu, H. (2022). Dielectric loss mechanism in electromagnetic wave absorbing materials. Adv. Sci. *9*, 2105553.
3. Zhang, K., Yan, Y., Wang, Z., Ma, G., Jia, D., Huang, X., and Zhou, Y. (2025). Integration of electrical properties and polarization loss modulation on atomic Fe–N-RGO for boosting electromagnetic wave absorption. Nano-Micro Lett. *17*, 46.
4. Wu, N., Liu, X., Zhao, C., Cui, C., and Xia, A. (2016). Effects of particle size on the magnetic and microwave absorption properties of carbon-coated nickel nanocapsules. J. Alloys Compd. *656*, 628–634.
